# I Don’t Know

**Published:** 1879-10

**Authors:** Thos. K. Beecher


### I DON'T KNOW.

BY THOS. K. BEECHER.

In a general way any man cheerfully admits that he don't know everything. But there are cases, when a man is supposed to know, expected to know, indeed his reputation and support depend on his seeming to know; and in all such cases it is hard for the man to say I don't know.

Indeed, it seems as if it were a man's duty, sometimes to conceal his ignorance and pretend to knowledge, for the sake of the comfort which a faith in him as a doctor or a teacher may be to his needy fellow-man. If a man set up as a horse doctor, and I bring him my limping beast, and ask what's the matter, he must answer something right away, or else confess that he knows no more than I do. I know such a horse doctor. He always has something to say. He will talk you blind as he fumbles over the old bones of a horse-leg in his shop, which I shrewdly guess he keeps as part of his impression machinery, or faith promoter. Now he is really a very useful man, but I don't believe that he knows half as much as he "lets on" to know.

I have no doubt that he is in the main a truthful man. Yet should I advise him always to say *I don't know*, when he don't know, it would be a piece of heroism and sacrifice little less than madness. For confidence is part of his capital. And who would employ a veterinary surgeon who kept saying, I don't know ?

Many a year ago when more than now I used to meet with teachers employed in our country schools I heard it suggested, don't ever say "I don't know." You must keep up the respect of your pupils. If you don't know it all, get round it some way till you have time to look it up.

Undoubtedly, children and the childish parents of childen have a false notion as to what makes a good teacher. They may think that any man that knows enough, is a good teacher; and if a man don't know, then he can't teach. As if knowledge in a man were like cider in a barrel. As if teaching were simply telling, or drawing cider. If this be true, of course, an empty barrel can give no cider, and a teacher that don't know can do no teaching. To satisfy the expectations of so-called patrons many a teacher falls into a stilted, imposing way both in and out of school.

Young ministers are afraid to say *I don't know*. To make such an answer seems like undermining one's own standing. What right has a man that don't know to stand in the pulpit ?

Skilled mechanics are subject to this same temptation. There are in this city three reasonably good timepieces, nay four. Every well-informed man knows that all clocks however costly have an error, greater or less. Not once in five years will those four timepieces agree to the second. Nor will it ever be possible by any amount of watchfulness to make any one of them a perfect timepiece. The best that can be done is to keep reasonably close to true time and closer still to regularity, and third, to display each day on the case of the timepiece the ascertained error—"two seconds slow," or "twelve seconds fast"—whatever it may be.

Yet I doubt whether any watchmaker in Elmira is courageous enough to put in big letters on his clock that it is ten seconds slow. Because the unthinking people would come in and say, "Well, that's a pretty clock. A nice standard. He is a queer watchmaker; runs his clock ten seconds slow and brags on it." The very fact that he publishes his error would show to intelligent men that he loved the truth and accuracy; and the attention of every man that is fit to carry a fine watch would be arrested. But in all probability it would repel more consulters than it would attract.

When a man has earned honestly a social position, a professional standing as a surgeon or a lawyer or a minister or a schoolmaster or a watchmaker, then perhaps he can "afford" to say I don't know. But until he has earned this position he is apt to feel that he cannot "afford" to do it. And very likely in the years during which he is too timid to tell the truth, and with more or less skill is keeping up appearances, there will happen some disaster of discovery, that will expose his ignorance and his falsity both; in which case, like any other liar he will fall as much lower than he professionally deserves as he was trying to boost himself higher.

This class of lies, that seems to be demanded by the unthinking and ignorant, is the hardest class to choke off. Teachers, preachers, and physicians are peculiarly liable to temptation as to this class.

For instance: In one of our famous theological schools, the perhaps foremost and most brilliant professor in these United States, gave counsel to his class somewhat thus:—

Young gentlemen, you will sometimes find yourself pushed in an argument, and hardly know what to say. There are many ways by which you can gain time to think. One of the best and one which I frequently use, is to say to your opponent, "please state your proposition again, I did not understand you." Very likely he will be confused by the demand. And so the professor went on to name several adroit dodges, by which the young minister would be able to escape the simple confession—I never thought of that before; or, that seems reasonable; or, I must take time to think; or any other true and modest reply, like *I don't know.*

One invariable earmark of a charlatan in medicine is, that he always knows and knows for certain the very moment anyone comes to consult him. And, on the other hand, one of the invariable characteristics of a man, fit to be trusted, is, that every now and then you will hear him say or hear of his saying, "I don't know, I shall have to watch this case, and read up and study a while yet, before I can make up my mind." Elderly physicians of established reputation may sometimes be heard so speaking. But young physicians would feel as if it would be their ruin; and perhaps it would for some months, or even years.

There are many hard sayings contained in scripture, which no man living can expound, nor any man that ever will live. There are many stubborn facts as to the administration of God in nature, and his proceedure as declared in various scriptures, that are difficult to reconcile—that never have been reconciled. And it is precisely at these spots where the intelligence of man stands gazing out into the infinitude of God, unable to shape the vision into any intelligible proposition—just here is where churches erect their *dogmas* and demand that men shall know and believe most positively. Whether they do know or not, they must say they know and say they believe. Thus in the high sanctuary of all our churches, the truth is murdered again and again.

The long and short then of what I am trying to say, is this: To love the truth and to speak the truth is a virtue which we all agree to praise But in those delicate relations between man and man which call for faith or trust amounting almost to reverence, it is peculiarly difficult for the man who is trusted ever to say, I don't know; or for a man who asks instruction to reverence his superior, all the more, for his truthfully saying, I don't know.

Nevertheless, in the long run, I am confident that he who loves the truth and pursues it regardless of consequences, will find after his costly sacrifices, and loss of patients and reputation, and custom, that he has gained a clearer vision to himself and increase of knowledge; and, by and by the tide will turn, and will roll back under him great tides of uplifting trust, such as no mere skill or tact can win.

